# Malocclusion in children with speech sound disorders and motor speech involvement: a cross-sectional clinical study in Swedish children

**DOI:** 10.1007/s40368-022-00728-4

**Published:** 2022-07-01

**Authors:** Å. Mogren, C. Havner, A. Westerlund, L. Sjögreen, M. Barr Agholme, A. Mcallister

**Affiliations:** 1 Division of Speech and Language Pathology, Department of Clinical Science, Intervention and Technology (CLINTEC), Karolinska Institutet, 141 86 Stockholm, Sweden; 2 Mun-H-Center, Orofacial Resource Centre for Rare Diseases, Public Dental Service, Gothenburg, Sweden; 3 Division of Orthodontic and Pediatric Dentistry, Department of Dental Medicine, Karolinska Institutet, Stockholm, Sweden; 4 Medical Unit Speech and Language Pathology, Women’s Health and Allied Health Professionals Theme, Karolinska University Hospital, Stockholm, Sweden; 5 Department of Orthodontics, Institute of Odontology, The Sahlgrenska Academy at Gothenburg University, Gothenburg, Sweden

**Keywords:** Multiprofessional assessment, Co-existing disorders, IOTN-DHC, Orofacial characteristics

## Abstract

**Objectives:**

The objectives of this study were to investigate the occurrence, types and severity of malocclusions in children with speech sound disorder (SSD) persisting after 6 years of age, and to compare these findings to a control group of children with typical speech development (TSD).

**Methods:**

In total, 105 children were included: 61 with SSD and motor speech involvement (mean age 8:5 ± 2:8 years; range 6:0–16:7 years, 14 girls and 47 boys) and 44 children with TSD (mean age 8:8 ± 1:6; range 6:0–12:2 years, 19 girls and 25 boys). Extra-oral and intra-oral examinations were performed by an orthodontist. The severity of malocclusion was scored using the IOTN-DHC Index.

**Results:**

There were differences between the SSD and TSD groups with regard to the prevalence, type, and severity of malocclusions; 61% of the children in the SSD group had a malocclusion, as compared to 29% in the TSD group. In addition, the malocclusions in the SSD group were rated as more severe. Functional posterior crossbite and habitual lateral and/or anterior shift appeared more frequently in the SSD group. Class III malocclusion, anterior open bite and scissors bite were found only in the SSD group.

**Conclusion:**

Children with SSD and motor speech involvement are more likely to have a higher prevalence of and more severe malocclusions than children with TSD.

## Introduction

The prevalence of malocclusion varies with subject age and across different populations. In Sweden, approximately 58% of 7-year-old children exhibit malocclusions (Dimberg et al. 2015). Occlusal development is mediated by both genetic and environmental factors. Growth patterns, muscle functions, breathing patterns, oral habits, and early tooth extractions are known to influence occlusal development (Linder-Aronson 1970; Ovsenik et al.^2007^). Prolonged sucking habits and residual orofacial dysfunction, such as incorrect tongue position (protruded tongue) and open mouth posture, have been strongly correlated to malocclusion in the primary and mixed dentition (Grabowski et al. 2007). From the ages of 3–12 years, non-nutritive sucking habits (NNS) and residual orofacial dysfunctions decline in children with typical development, with a decrease also seen in the number of functional malocclusions (Ovsenik et al. 2007). However, this is not always the case in children with neurodevelopmental disorders (NDD) and orofacial dysfunction (Yogi et al. 2018). Malocclusion has been described as a common finding in individuals with NDD (de Castilho et al. 2018; Fontaine-Sylvestre et al. 2017; Miamoto et al. 2010; Vellappally et al. 2014) and is particularly prominent when orofacial dysfunction is present (Sjogreen et al. 2015a, b).

Other defects of the orofacial morphology, such as tongue-tie (ankyloglossia) (Pompéia et al. 2017) and tonsil hypertrophy (Hultcrantz et al. 1991; Lundeborg et al. 2009; Valera et al. 2003), can influence both occlusal development and orofacial functions, including speech, swallowing, nose breathing and voice quality (Lundeborg et al. 2012). Hyperactivity of the *musculus mentalis (m mentalis)* is related to malocclusion, especially for Class II bite (Pintavirooj et al. 2014). Hyperactivity of the m mentalis and the inferior part of the lower lip has been reported as not only a consequence of malocclusion and incompetent lip closure (Jung et al. 2010; Yamaguchi et al. 2000), but also as a contributing factor to the development of malocclusion and orthodontic relapse (de Souza et al. 2008; Lee et al. 2021).

Speech sound disorders (SSD) are common in developing children and regarded as a NDD (Shriberg, 2010). Children with SSD form a heterogeneous group and differ with respect to the underlying cause and severity of the speech difficulty (Namasivayam et al. 2019; Waring and Knight 2013). The aetiology can be idiopathic or related to a known genetic or acquired diagnosis (Newbury and Monaco 2010). Between 2 and 13% of children in the age range of 6–8 years are reported to have SSD depending on the applied definition of the disorder (Shriberg et al. 1999; Wren et al. 2016).

In earlier studies of the relationship between SSD and malocclusion, the focus was on how different malocclusions interfere with speech sound production (Laine 1987, 1992; Leavy et al. 2016). Specific speech sounds have been linked to specific types of malocclusions. Structural deviations, especially those in the anterior part of the oral cavity, can interfere with lip and tongue placement for speech sounds (Jensen 1968; Laine 1987; Subtelny et al. 1964). An anterior open bite (AOB) can result in interdental production of dental fricatives (e.g. /s/), and the articulation of labio-dental fricatives (/f/, /v/) can be affected by a Class III occlusion (Profitt 2013). However, speech is a complex cognitive and motor activity with specific requirements for precision and neurological control (Moore and Ruark 1996). Koskela et al. (2020) have reported that children with severe malocclusions have speech difficulties more often than control subjects. However, they concluded that this might reflect a shared genetic aetiology rather than a causal relationship.

Concomitant occurrence of orofacial dysfunction has been observed in children with speech and language disorders of different aetiologies (Bishop 2020; Hill 2001; Mogren et al. 2020; Visscher et al. 2007). Orofacial dysfunction can influence the development of occlusion (D'Onofrio 2019; Kiliaridis et al.^1995^; Sjogreen et al. 2015a, b; Stahl et al. 2007). The hypothesis underlying the present study is that children with SSD have more malocclusions than children with typical speech development (TSD).

The aims of this study were to investigate the occurrence, types and severity of malocclusion in a group of children with SSD persisting after the age of 6 years and to compare these findings to a control group of children with TSD. An additional aim was to determine if and how oral habits and other orofacial characteristics (tongue-tie, enlarged tonsils and hyperactivity of the m mentalis) are related to malocclusion.

## Subjects and methods

### Participants

The study included a group of children with SSD and a control group of children with TSD.

#### SSD group

The SSD group consisted of 61 children (mean age 8.5 ± 2.8 years; age range 6.0–16.7 years), including 14 girls and 47 boys. The participants were consecutive patients with SSD referred to an orofacial resource centre for speech and oral motor examinations during the period 2014–2016.

The inclusion criteria were SSD persisting after the age of 6 years; a lack of moderate or severe intellectual disability; and cerebral palsy and/or severe autism spectrum disorder (ASD). Sixty-two consecutive patients who met the inclusion criteria were offered to participate in the study, all but one patient accepted participation.

Nine participants reported a confirmed NDD other than SSD, such as ADHD or ASD. Five participants were raised in bilingual homes but had Swedish as their first language and two children were adopted internationally at 2:6 and 3 years of age. Three sibling pairs were included. All participants but one followed the regular curriculum for compulsory schooling.

The children with SSD had speech difficulties to varying degrees. They were all clinically assessed by a speech-language pathologist as having a motor speech disorder. Twenty-five children were assessed as having Childhood Apraxia of Speech (CAS), 23 children had Speech Motor Delay (SMD), 9 had SMD/ suspected CAS, 3 exhibited articulation impairment, and 1 had Developmental Dysarthria. All the children with SSD showed impaired consonant production and no single consonant was established in all children. The mean percentage consonants correct (PCC) was 66% (± 22.1), as compared to the expected 97.8% for typically developing 7-year-old Swedish children and 99.4% for typically developing 19-year-olds (Lohmander et al. 2017). PCC is a well-established way to measure the severity of SSD (Shriberg et al. 1997). Only 13% of the participants had an established production of /r/, 29% of /s/, and 33% of /l/ (Mogren et al. 2020).

Orofacial function had been assessed using Nordic Orofacial Test-Screening (NOT-S) (Bakke et al. 2007). This test consists of an interview part and an examination part, with six domains in each part. The maximum NOT-S score is 12, with one score for each domain. Typically developing children under the age of 5 years have a mean score of < 2 (McAllister and Lundeborg 2013). Thus, a result ≥ 2 is regarded as orofacial dysfunction. Based on the results of the NOT-S, 87% of the children in the SSD group displayed difficulties with orofacial function (total score for NOT-S ≥ 2) (Mogren et al. 2020). The mean NOT-S score in the SSD group was 4.0 ± 2.2.

#### TSD group

The TSD group consisted of 44 children (mean age 8.8 ± 1.6; age range 6.0–12.2 years), including 19 girls and 25 boys. The children in the TSD group were consecutively recruited after advertising at the Public Dental Health Service where the children had their regular dental care. The inclusion criteria were typical speech development; age between 6 and 18 years; and no known NDD. In the TSD group, 98% had a score of 0 (78%) or 1 (20%) on the NOT-S test, indicating no orofacial dysfunction. Only one child (2%) had a NOT-S result ≥ 2. All the NOT-S scores in the TSD group were obtained in the interview part of the test. The mean NOT-S score in the TSD group was 0.2 ± 0.5.

### Procedure and test items

A speech-language pathologist and an orthodontist performed the data collection and all the assessments in a clinical dental setting. A set of intra-oral and extra-oral photographs was acquired during the clinical examination and used to assess the inter- and intra-reliability levels. A questionnaire that contained items related to present and previous oral habits and orthodontic treatment was filled in by the parents of the participants. Extra-oral and intra-oral examinations were performed by an orthodontist (CH). Assessments of tonsils, tongue-tie and m mentalis were performed in consensus agreement with a Speech-Language Pathologist.

#### IOTN index

The scores on the IOTN-DHC (Index of Orthodontic Treatment Need, Dental Health Component) Index (Brook and Shaw 1989), malocclusion (yes/no), and type of malocclusion were assessed on intra-oral and extra-oral photographs by two orthodontists (CH, AW). The IOTN-DHC (hereinafter referred to as IOTN) was used to describe the orthodontic treatment need in the groups and also to describe the severity of the malocclusions according to the index’ priority. The malocclusions were scored from 1 to 5. As no x-radiographs were taken, hypodontia, hyperdontia and ectopic eruption or impacted teeth could not be examined or included in the assessment. Participants with IOTN grades 1 (almost perfection), 2 (minor irregularities) or 3d (moderate space anomalies) were not considered to have a malocclusion. The exception to this was 2c, which describes minor functional anterior or posterior crossbites and bilateral crossbites without shift. Participants with IOTN grades 2c, 3abcef, 4 and 5 were considered to have a malocclusion. Functional crossbite was assessed as a primary interference in retruded mandibular position resulting in a lateral or anterior shift of the mandible with at least one segment (≥ 3 occlusal pairs) crossbite. The reason for excluding moderate space anomalies was to minimise the number of false-positive malocclusions in young individuals with crowding during the transition to permanent dentition, a condition considered a normal finding to some extent in occlusal development.

### Statistical analysis

All the data were analysed using the Statistical Package for the Social Sciences (IBM SPSS Statistics ver. 22.0 software). The level of significance was set at *p* < 0.05 throughout. Descriptive statistics were used for the background features, age and sex of the participants. When the data were nominal, Pearson’s Chi-squared test was used for between-group comparisons, and when the data were ordinal the Mann–Whitney *U* test was used. The *Pearson* correlation coefficient (Pearson’s *r*) was used for the correlation analysis of age with IOTN. The levels of inter- and intra-rater agreement of the IOTN and type of malocclusions were calculated using Cohen's *kappa coefficient*. Inter-rater agreement re-assessments were made for all participants. Intra-rater agreement re-assessments were made for 20% of the participants, selected randomly.

## Results

### Parent-reported information

None of the participants in either the SSD group or the TSD group used a pacifier or engaged in thumb sucking at the time of the assessment, although 19 participants (31%) in the SSD group reported a current oral habit, such as nail biting (*N* = 10), biting the lips (*N* = 11) or teeth grinding during daytime (*N* = 5). In the TSD group, nail biting was the only reported habit for eight participants (18%). There were no differences in earlier NNS between the children in the SSD group and the children in the TSD group. In the SSD group, 19% (8/41) continued with an NNS habit after 3 years of age and the corresponding percentage in the TSD group was 21% (9/43). Three children in the SSD group (9:4 years, 11:2 years and 16:8 years of age) had ongoing or previous orthodontic treatment.

### Occurrence and types of malocclusions

The children with SSD had more malocclusions than the children with TSD (Table 1). Furthermore, there were differences between the two groups with regard to the types of malocclusions. Functional posterior crossbite was more common in the SSD group (21%) than in the TSD group (2%). A high number (21%) of the children in the SSD group also exhibited a pattern that involved habitual lateral and/or anterior shift (HLAS) of the mandible during speech and rest position, such that the child had difficulties finding the intercuspal position, even when tactile guidance was given from the examiner. In the TSD group, only 2% of the children had this HLAS pattern (*p* = 0.005).

**Table 1:** Rates of occurrence and types of malocclusions in children with speech sound disorders (*N* = 61) and in children with typical speech development (*N* = 44)

Type of occlusion	Speech sound disorders % (*N*)	Typical speech development % (*N*)	*p *value^1^
Malocclusion in total	61 (37)	27 (12)	** < 0.001**
Class II (postnormal)	25 (15)	18 (8)	0.433
Class III (prenormal)	15 (9)	0	**0.008**
Deep bite	23 (14)	4 (2)	**0.010**
Anterior open bite	12 (7)	0	**0.020**
Scissors bite	3 (2)	0	0.225
Posterior crossbite (non-functional)	0	4 (2)	0.123
Functional posterior crossbite	21 (13)	2 (1)	**0.005**

^1^The *p *values are derived from Pearson’s Chi-squared test. Statistically significant values are bolded

The Class III relation, anterior open bite (AOB), and scissors bite were detected only in the SSD group. Class II relation was the most common malocclusion in both the SSD group and the TSD group. Deep bite (23%) was almost as common as Class II relation (25%) in the SSD group, whereas deep bite was not common at all (4%) in the TSD group. In the SSD group, the frequency of Class I occlusal relation was 61% and in the TSD group it was 82%. The three children with ongoing or previous orthodontic treatment still had a malocclusion. One had a deep bite, while the other two had functional anterior and posterior crossbites. These three children were not excluded from the group since their malocclusion was still present. Regarding the age heterogeneity of the participants, there was no correlation between the occurrence of a certain type of malocclusion and age, neither in the SSD group nor in the TSD group.

### Severity of malocclusion according to IOTN

There was a difference in IOTN grades between the SSD group and the TSD group (Table 2). The median IOTN value for the SSD group was 3 and for the TSD group it was 2 (*p* = 0.001). No correlations were found between age and IOTN grade in the group as a whole or in the separate groups (SSD and TSD). There were three participants with IOTN 2c (two with non-functional posterior crossbite and one with functional posterior crossbite) who were considered as having a malocclusion despite IOTN 2, all of them in the TSD group.

**Table 2:** Orthodontic treatment need and severity of malocclusion graded with the IOTN-DHC index in children with speech sound disorders (*N* = 61) and in children typical speech development (*N* = 44)

IOTN index grade	Speech sound disorders % (*N*)	Typical speech development % (*N*)
Grade 1–2	38 (23)	79 (35)
Grade 3	26 (16)	7 (3)
Grade 4	36 (22)	14 (6)

### Oral habits and previous NNS behaviour in relation to different malocclusions

There were no differences in earlier NNS behaviour between the children with or without malocclusion (in both the SSD group and TSD group) (Table 3). Children with a Class II malocclusion had more ongoing oral habits than children with other types of malocclusion and children with no malocclusions (*p* = 0.027).

**Table 3:** Reported present oral habits and previous non-nutritive sucking habits in relation to malocclusions in all children (*N* = 105)

	Present oral habits % (*N*)	Previous non-nutritive sucking habits ≥ 3 years % (*N*/*N*)^a^
No malocclusion, *N* = 56	29 (16)	23 (11/47)
Class II, *N* = 23	43 (10)^b^	29 (5/17)
Class III, *N* = 9	40 (2)	20 (1/5)
PC, *N* = 16	19 (3)	7 (1/12)
Deep bite, *N* = 16	25 (4)	0 (0/8)
AOB, *N* = 7	14 (1)	17 (1/6)

Children with speech sound disorders (*N* = 61), children with typical speech development (*N* = 44)

*PC* posterior crossbite, *AOB* anterior open bite

^a^Due to missing data on previous non-nutritive sucking habits the first *N* stands for the number of reported previous non-nutritive sucking habits and the second for the number of participants with the malocclusion that answered the question

^b^*p* = 0.027, according to Pearson’s Chi-squared test

### Orofacial characteristics

More children in the SSD group had a tongue-tie than in the TSD group (Table 4), although the difference was not significant. There was no significant difference between the groups regarding enlarged tonsils, whereas hyperactivity of the m mentalis was significantly more common in the children with SSD (Table 4). Enlarged tonsils were somewhat more common in the children with SSD and TSD with posterior crossbite (both non-functional and functional), although the difference was not significant (Table 5). More children with AOB had enlarged tonsils and hyperactivity of the m mentalis (Table 5).

**Table 4:** Orofacial characteristics of the children with speech sound disorders (*N* = 61) and of the children typical speech development (*N* = 44)

Variable	Speech sound disorder % (N)	Typical speech development % (N)	*p* value^1^
Tongue-tie	11 (7)	2 (1)	0.079
Enlarged tonsils	23 (14)	18 (8)	0.554
Hyperactivity of the m mentalis	31 (19)	7 (3)	**0.002**

^1^The *p v*alues are derived from Pearson’s Chi-squared test. Statistically significant values are bolded

**Table 5:** Orofacial characteristics in different malocclusions in all children (*N* = 105)

	Tongue-tie % (*N*)	Enlarged tonsils % (*N*)	Hyperactivity of m mentalis % (*N*)
Class II, *N* = 23	9 (2)	22 (5)	35 (8)
Class III, *N* = 9	11(1)	22 (2)	33 (3)
PC, *N* = 16	6 (1)	37.5 (6)	19 (3)
Deep bite, *N* = 16	0 (0)	6 (1)	31 (5)
AOB, *N* = 7	14 (1)	57 (4)*	71 (5)**

Children with speech sound disorders (*N* = 61), children with typical speech development (*N* = 44)

*PC* posterior crossbite, *AOB* anterior open bite

*p = 0.015, **p = 0.001, according to Pearson’s Chi-squared test

### Intra- and inter-rater agreement levels

The inter-rater reliability in relation to the IOTN index was calculated with Kappa statistics and was estimated to be good (*κ* = 0.706) (Altman 1991). Inter-rater agreement as to the type of malocclusion was slightly lower but still judged to be good with 83% point-by-point agreement. The intra-rater reliability in relation to the IOTN index was estimated to be very good (*κ* = 0.901). The intra-rater agreement as to the type of malocclusion was also very good with 95% point-by-point agreement.

## Discussion

The prevalence of malocclusion was high in the children with SSD and motor speech involvement, as compared with a control group of children with TSD. There were significant differences between the groups regarding the overall prevalence, type, and severity of malocclusions. This is in agreement with the results of previous studies on malocclusions in children with NDD (de Castilho et al. 2018; Fontaine-Sylvestre et al. 2017; Miamoto et al. 2010; Sjogreen et al. 2015a, b; Vellappally et al. 2014). The results obtained herein are not interpreted as indicating a causal relationship between occlusion and speech. It is more likely that the development patterns of occlusion and speech are influenced by innate motor function development and congenital orofacial features, and that these symptoms have the same biological background. However, it seems unlikely that the deficits seen in all of the children with SSD in the present study can be attributed to having the same genetic background.

Certain types of malocclusions were observed more frequently in the children with SSD than in the children with TSD. The prevalence of crossbite is estimated to be 8.5–17.0% for typically developing children (Bässler-Zeltmann et al. 1998; Heikinheimo 1978; Perillo et al. 2010; Thilander and Myrberg 1973). The international literature does not usually separate functional crossbite from non-functional, which makes direct comparisons difficult. In this study, there was a difference between children with SSD and children with TSD regarding functional posterior crossbite, which is in line with the more muscle-related difficulties seen in the SSD group. All posterior crossbites in the SSD group were functional. A higher percentage of posterior crossbites has been reported in children with ASD (Fontaine-Sylvestre et al. 2017). Those authors related this finding to a higher frequency of oral habits in the ASD group. The children with SSD in the present study reported somewhat more oral habits, although this only partially explains the differences between the groups. The children with functional posterior crossbite did not show a higher frequency of oral habits than the children with other malocclusions and they had not used a pacifier for a longer time than the other children. An alternative explanation for this difference could be the observed imbalance in muscle function and jaw instability.

During the dental assessment, many of the children with SSD exhibited difficulties with finding their intercuspal position. They had a jaw-gliding/“groping” pattern that represented a habitual lateral and/or anterior shift of the jaw. Jaw instability has been reported in children with SSD and orofacial dysfunction (Mogren et al. 2020, 2021; Namasivayam et al. 2013). Control and stability of the jaw is a prerequisite for controlled movements of the lips and tongue (Wilson and Nip 2011), and lack of jaw stability and control may be common also in children who have milder orofacial dysfunctions. A longitudinal study of children who exhibit HLAS is needed to resolve this issue. Younger children often show a jaw instability profile when asked to bite together during a dental examination. Younger children have more flexible joints and less-mature oral motor skills, which can have a negative effect on jaw stability. Nevertheless, the participants with SSD in this study were too old to be expected to maintain this jaw stability immature pattern. The highly significant difference between children with SSD and TSD regarding jaw stability is noteworthy and in line with earlier observations of children with SSD (Namasivayam et al. 2013). Children with the motor speech disorder CAS are often described as exhibiting a “groping” behaviour (Chenausky et al. 2020), in that they have a pre-articulatory muscular seeking pattern. The jaw gliding (HLAS) observed in the present study could be interpreted as a groping behaviour. To compensate for the lack of stability and control of the jaw/mandible, some children develop a fixing pattern, whereby they clench their teeth together to lock the jaw in a fixed closed position and keep the lips retracted. This fixing pattern has been suggested to act as a compensatory mechanism to stabilise the jaw and allow the lips and tongue to move more freely (Ward et al. 2013). This could result in clenched speech articulation. It might also explain why the deep bite feature was significantly more common in the SSD group.

The Class III relation existed only in the SSD group. The prevalence of Class III relations in Swedish schoolchildren (7–13 years of age) is estimated to be 6% (Thilander and Myrberg 1973). The percentage of children in the SSD group who had a Class III relation was only slightly higher at 8%. Nevertheless, no Class III relation was found in the control group. Class III relations can develop due to either deficient growth of the maxilla or excessive growth of the mandible (De Clerck and Proffit 2015). Class III relations are attributed primarily to heredity (Profitt 2013). This craniofacial trait in the SSD group in the present study may represent a minor morphological deviation without medical significance, which is sometimes seen in children who have an NDD and have a specific genetic background (Ozgen et al. 2011). Class III relations are expected to be more prevalent in older individuals due to the growth of the mandible in late adolescence, although this was not the case in this study.

AOB was only observed in the SSD group. Castilho and colleagues (de Castilho et al. 2018) have identified open mouth posture and use of a pacifier as risk factors for developing AOB in children with developmental disorders. In the present study, we did not find any relationship between on-going or former oral habits and AOB.

Class II relation was the most common malocclusion in both the SSD group and the TSD group. It was somewhat more common in the SSD group, although the difference was not significant. Class II relation has been reported as the most common malocclusion in mixed dentition (Lombardo et al. 2020). The children with Class II relations in this study were more prone to have an ongoing oral habit, which is in line with the findings of previous studies (Baeshen 2021; Grippaudo et al. 2016).

Sjogreen et al. (2015a, b) have shown that individuals with rare diseases have a higher prevalence and increased severity of malocclusion, as compared to controls with typical development. They have also reported a difference in occlusion between participants with and without oromotor impairment. Class II malocclusion, Class III malocclusion, AOB, and deep bite were all found to be more common in individuals with oromotor impairment (Sjogreen et al. 2015a, b). As in the present study, the underlying aetiology has not been fully established, but may be linked to a combination of genetic and environmental factors. Nevertheless, orofacial dysfunction seems to play an important role in occlusal development and co-exists in individuals with NDD and malocclusion, lending support to the findings of the current study.

The children with SSD exhibited a more severe malocclusion than the children with TSD, as assessed with the IOTN Index. The order of priority of the IOTN Index follows the principle of “MOCDO” (missing, overjet, crossbite, displacement, overbite). In this study, no radiological examination was performed, so none of the participants were assessed as having the highest score due to missing or impacted teeth (Yogi et al. 2018). Increased overjet, which has a high priority in the Index, was the most prevalent malocclusion in both groups. The children with SSD displayed more severe sagittal and vertical deviations, as well as functional malocclusions than the children with TSD. While the reason for this difference needs to be explored further, it may be connected to genetics and the higher incidence of orofacial dysfunctions in the SSD group.

Oral habits or earlier NNS behaviour were not more common in children with malocclusion in this study, except for children with a Class II malocclusion for whom it was somewhat more common to have an ongoing oral habit. This is in line with the results from a longitudinal study conducted by Dimberg et al. (2015), in which no association between sucking habits and malocclusion was found in typically developing children with malocclusions who were 3–11.6 years old.

Hyperactivity of the m. mentalis was far more common in the SSD group and especially common in children with AOB. This could be interpreted as a compensatory fixing pattern to stabilise the earlier-described jaw instability, through the recruitment of an additional muscle unit to maintain the jaw in position and achieve lip closure. Previous reports have confirmed the close relationship between activation of the m mentalis and a Class II malocclusion, as well as in AOB (Pintavirooj et al. 2014). The m. mentalis stabilises the lower lip. This allows lip closure when the distance is increased due to malocclusion, increased lower facial height or a short/retracted upper lip. It can also be a sign of lip hypofunction. In a study conducted by de Souza et al (2008), Class II:1 patients with incompetent lip closure displayed essentially the same level of m. mentalis activation before and after orthodontic correction with premolar extraction, indicating that the underlying dysfunction had not been addressed. Enlarged tonsils were also more common in children with AOB, which is in line with the aforementioned relationship between AOB and open mouth posture (Grabowski et al. 2007). Tongue-tie was somewhat more common in the children with SSD but did not show any relationship with any of the malocclusions in this study. This is in line with an earlier study by Sepet et al. (2015) where they did not find any relation between mild and moderate ankyloglossia and occlusion type. However, in a study by Vaz and Bai (2015), they found a relation between tongue-tie and AOB.

There is a high frequency of co-existing symptoms in children with NDD, such as SSD (Gillberg, 2010). The speech disorder is often the symptom that has the greatest impact on overall function in daily life. For the clinician, it is important to be observant of all symptoms that might influence the patient’s quality of life, such as malocclusions and chewing difficulties, to make adequate referrals for improved diagnostics and care. Malocclusions have been reported as possibly having negative effects on emotional and social well-being (Dimberg et al. 2016), and impaired chewing ability has been associated with reduced oral health-related quality of life in adults (Brennan et al. 2008).

Children with NDD are also at higher risk of having facial morphological traits (Ozgen et al. 2011). This is another reason why it is important to follow occlusal development in this population.

There are some limitations in the study that should be considered by future researchers. The children in the SSD group were all consecutive patients referred to the orofacial resource centre for a speech and oral motor examination which may have influenced the results. In other words, the exact frequency of malocclusion found in our group of children with SSD may not be fully representative for the patient group as a whole. The children in the TSD group were recruited from the Public Dental Health Service which offers dental care to all Swedish children. The children in the TSD group are in this way representative for the age group, but according to the inclusion criteria, the control group should not have any NDD; thus, our group may present less co-existing symptoms which could result in less and less severe malocclusions. Children between the ages of 6 years and 9 years 11 months were more strongly represented in both the SSD group and the TSD group, reflecting a limitation of the study. A more-even age distribution would have been preferable. On the other hand, no relationships were noted between the different malocclusions and age and no malocclusion type was found to be more common in the younger or older age groups.

This study adds knowledge regarding the features of occlusal and dento-facial development that can be affected in children with milder NDD of unknown origin, such as SSD. The children with SSD in the present study did not have severe orofacial dysfunction, severe gross motor dysfunction, intellectual disabilities or severe autism. The high incidence of malocclusion noted in the SSD group highlights the importance of dental professionals and Speech-Language Pathologists being alert to co-existing malocclusions in children with SSD and NDD, to facilitate orthodontic intervention or correction. This study indicates a shared underlying biological cause between SSD and malocclusion and emphasises the need for a holistic and multi-professional approach when screening for co-existing symptoms in children with SSD (NDD).

## Conclusion

Children with SSD and motor speech involvement are more likely to present with malocclusions and have more severe malocclusions, as compared to children with TSD. The malocclusions found in children with SSD, such as functional crossbite and anterior open bite, may be related to orofacial dysfunctions. Hyperactivity in m mentalis was more common in children with SSD and specifically related to AOB. The co-existing malocclusions in children with SSD underline the importance of addressing occlusal development and describing the orofacial features in children with SSD. This should provide valuable knowledge when describing the phenotype in clinical genetic investigations.

## Data Availability

The authors confirm that the data supporting the findings of this study are available within the article. The data will be shared on reasonable request to the corresponding author.
